# Breast cancer and COVID-19: The need for enhanced psychological support for women with breast cancer during the pandemic

**DOI:** 10.18332/ejm/156899

**Published:** 2022-12-22

**Authors:** Dimitrios Charos, Maria Andriopoulou, Victoria Vivilaki

**Affiliations:** 1 Department of Midwifery, University of West Attica, Athens, Greece; 2 General Anticancer- Oncology Hospital ‘Agios Savvas’, Athens, Greece; 3 General Hospital of Nea Ionia ‘Konstantopouleio’, Athens, Greece

**Keywords:** COVID-19, breast cancer, psychological impact, psychological support

**Dear Editor,**

Statistical estimates show that the coronavirus pandemic affects cancer^1^. Reports in various countries, such as the Netherlands, the United Kingdom, and the USA, showed that during the pandemic there were fewer breast cancer diagnoses while cancer mortality continued to increase^2,3^. At the same time, screening was reduced or temporarily discontinued, patients had difficulty accessing and ensuring their care and prevention, delaying or postponing surgery, all of which had a significant impact on the care of women with cancer^2,3^.

The COVID-19 pandemic is a highly stressful event for breast cancer women due to the vulnerability they feel from the disease itself, the possibility of contracting the coronavirus, but also from the conditions created by the pandemic^4,5^. Breast cancer women due to immunosuppression are at increased risk of developing COVID-19 complications compared to the general population^6^. The restrictive measures had a significant psychological impact on women with cancer, which affected them socially and reduced social support^6^. Evidence from studies in countries such as Canada, the United Kingdom etc., shows that postponing surgeries, interruptions, delays and changes in their treatment due to overload of the health system have had an impact on women and increased anxiety, distress, depression, insomnia, fear of cancer recurrence, emotional distress, and emotional vulnerability associated with the pandemic^4,5,7-11^.

The specific population with breast cancer is particularly vulnerable to developing emotional disturbance during a pandemic. In the United Kingdom, job insecurity caused by the pandemic crisis has increased stress, emotional distress and depression in women already suffering from breast cancer, and has also affected their cognitive function^10^.

In many hospitals during the pandemic, caregivers’ visits to patients were very limited and at times visits banned, leading patients to social isolation and frustration from being prevented to be visited by relatives^5^. In addition, the strict confinement in their homes reduced social contacts, increasing feelings of social isolation and resignation, while making it difficult for many families to express their feelings, and in the case of mourning to do this as they would have done normally^5^.

It is also worth noting that the COVID-19 pandemic has negatively affected the community midwives and other health professionals who care for breast cancer patients^8,12^. Health professionals and the community midwives experienced more stress, insecurity and exhaustion, which affected their functionality and their quality of life^8,12^.

The impact of the pandemic on women with breast cancer was significant and increased patients’ vulnerability, emotional distress, depression, and anxiety^4^. Therefore, it is necessary to have a different support framework for breast cancer women during the pandemic crisis that will maintain their care^5^. Online interventions are valuable and can be effective in managing the stress and emotional distress of breast cancer women^4,5,10,11^. Web conferencing can provide psychological support, reducing emotional distress, fear, social isolation, stress and the accompanying effects of cancer during the pandemic^4,11^. Similarly, telephone supportive care has proved very useful5,8. In addition, the contribution of online support groups is important in increasing social support, helping to deal with vulnerability, and increasing quality of life^4,5,11^.

In conclusion, the COVID-19 pandemic crisis has placed a further burden on breast cancer women. The need to support women with breast cancer is more urgent than ever and can, in part, be effectively achieved online. Communication with and support for women with breast cancer through digital media or telephone by the community midwives and other health professionals, would be very valuable during the pandemic.

## Data Availability

Data sharing is not applicable to this article as no new data were created.
